# Facing Up to Unpalatable Evidence for the Sake of Our Patients

**DOI:** 10.1371/journal.pmed.1000112

**Published:** 2009-08-18

**Authors:** Paul E. Mullen

**Affiliations:** 1 Monash University, Melbourne, Victoria, Australia; 2 Institute of Psychiatry, London, United Kingdom

## Abstract

Paul Mullen discusses Seena Fazel and colleagues' paper on the association between violent behavior and having been diagnosed with a schizophrenic disorder, and its implications for care of these individuals.

**Linked Research Article**

This Perspective discusses the following new study published in *PLoS Medicine*:

Fazel S, Gulati G, Linsell L, Geddes JR, Grann M (2009) Schizophrenia and violence: Systematic review and meta-analysis. PLoS Med 6(8): e1000120. doi:10.1371/journal.pmed.1000120

Seena Fazel and colleagues investigate the association between schizophrenia and other psychoses and violence and violent offending, and show that the increased risk appears to be partly mediated by substance abuse comorbidity.

The paper of Seena Fazel and colleagues published in this week's *PLoS Medicine* [1] provides the most extensive and sophisticated analysis to date of the available data on the association between violent behaviour and having been diagnosed with a schizophrenic disorder. The possible association between schizophrenia and violence remains a contentious issue in mental health. This question is particularly emotive for those, like myself, who started their psychiatric careers at a time when massive asylums still dominated the landscape of mental health care, or the lack of care, and the struggle for civil rights for the compulsorily detained was just beginning. Those working for reform confronted the necessity of calming the exaggerated fears of the general population about the violent tendencies of the mad. Equally it was essential to overcome similar, though more politely articulated prejudices among those who controlled public mental health services, including most of our older colleagues. The question of an association was in those days as much a political as a scientific question, and it was in the guise of scientists that we answered politically [2]–[4]. The efforts to minimise, or if possible explain away, the apparent association between schizophrenia and violence was remarkably effective, and up to a point beneficial to patients. Several generations of mental health professionals were taught there was no association, patient advocacy groups gratefully accepted the new wisdom, and even journalists and politicians became somewhat more constrained in evoking the fear of the murderous mad.

But in the end the question deserves to be answered empirically, both for our own scientific integrity, and far more importantly for the sake of our patients. If the association is wrongly dismissed nothing can be done to reduce the risks of possible violence, with its attendant disasters for victim and patient. Currently many mental health professionals refuse to accept that the reduction of the violence potential in their psychotic patients is any of their business. Worse, the emergence of aggressive and threatening behaviour can sometimes lead to a reduced level of care, or even a withdrawal of services. This is occasionally justified by a reclassification from schizophrenia to borderline or antisocial personality disorder (depending on gender).

The review by Fazel and his colleagues provides the evidence base for a relationship between having a schizophrenic syndrome and behaving violently. The level of the association is not trivial, being as compared to the general population, four to five times greater for general violence, and between 14 and 25 for homicide. The review also makes clear that substance abuse is a major risk factor for violence in schizophrenia.

Associations and risk factors are statistically significant correlations; they do not establish causality. At best they demonstrate a rebuttable presumption of causality. Having established a correlation the question immediately shifts to whether it is a result of confounding factors, and if not how it is caused and mediated.

In the more recent literature reviewed by Fazel and colleagues substance abuse is often implied to be a confounder establishing an apparent connection to schizophrenia, but in fact being an independent causal factor largely unrelated to the illness itself [5]. Substance abuse as the major or sole cause of the violence has considerable appeal. This explanation is consonant with current popular and politically expedient explanations of crime in general. It transfers not only cause but the stigma to drug and alcohol abuse. Finally, it both shifts responsibility away from mainstream psychiatric services to substance abuse services (separate in many jurisdictions), and offers a clear management strategy. Such a welcome escape from the potentially difficult implications of the research reviewed here, however, needs cautious consideration.

Substance abuse currently is prevalent among those with a schizophrenic syndrome, and is often well established at first presentation. The propensity to abuse substances and to behave violently could spring independently from common factors related to aspects of the schizophrenic syndrome. Another possibility is that the social decline and disorganisation so often the fate of the seriously mentally ill may underlie the turn to intoxicants. These possibilities would imply that substance abuse either mediates between aspects of the core schizophrenic syndrome and violent behaviour, or is essentially independent of the propensity for violence. The latter radical suggestion can find some support in the literature. Studies that examined rates of violence in those with schizophrenia over time suggest violence rates have remained relatively stable despite substance abuse rates escalating [6],[7]. Similarly, Fazel and colleagues' review suggests that rates of violence have not increased over the last 38 years, despite the known and dramatic increase in comorbid substance abuse.

To relegate substance abuse to an epiphenomenon with regards to violence and schizophrenia would be as premature and foolish as to assume that substance abuse is the complete causal explanation. The middle ground better reflects the current state of knowledge. Substance abuse in and of itself increases the social and clinical disorganisation of the patient, which in turn makes antisocial behaviour more likely. The increased rates of substance abuse in schizophrenia reflect both core features of the syndrome and the social disadvantage consequent on rejection and remediable disability. Finally, there are probably factors in some forms of schizophrenia that affect the ease with which aggression is evoked and reduce the inhibition of violent responses.

Accepting the association between schizophrenia and violent behaviour is the first stage for any meaningful clinical response. The reduction of that violence requires, in my opinion, good mental health care. Admissions, particularly first admission, should no longer be of a few days, but of adequate length to properly assess the patient, bring their active symptoms under reasonable control, dry them out from drugs and alcohol, and establish a good enough therapeutic relationship. A multidisciplinary team is required for subsequent management in the community, with one clinician providing continuity, support, and active maintenance of contact. A focus on the patient's active symptoms is insufficient. There also needs to be management of the substance abuse, the social isolation and dislocation, the criminogenic personality traits, and a reengagement in economic and social roles. Such a recipe may seem idealistic and absurdly costly, but for a measure that could decrease homicide by up to 8% and all violent crime by 4% it would be a bargain. Just think of the cost in financial and human terms of serious violence and homicide. In addition, it would provide one of the most disadvantaged groups of patients with decent care for once in our history.

## References

[1] Fazel S, Gulati G, Linsell L, Geddes JR, Grann M (2009). Schizophrenia and violence: Systematic review and meta-analysis.. PLoS Med.

[2] Häfner H, Böker W, Marshall FH (1982). Crimes of violence by mentally abnormal offenders..

[3] Monahan J (1981). The clinical prediction of violent behaviour..

[4] Mullen PE (1984). Mental disorder and dangerousness.. Aust N Z J Psychiatry.

[5] Steadman HJ, Mulvey EP, Monahan J, Robbins PC, Appelbaum PS (1998). Violence by people discharged from acute psychiatric inpatient facilities and by others in the same neighborhoods.. Arch Gen Psychiatry.

[6] Wallace C, Mullen PE, Burgess P (2004). Criminal offending in schizophrenia over a 25-year period marked by deinstitutionalization and increasing prevalence of comorbid substance use disorders.. Am J Psychiatry.

[7] Vevera J, Hubbard A, Veselý A, Papezová H (2005). Violent behaviour in schizophrenia. Retrospective study of four independent samples from Prague, 1949 to 2000.. Br J Psychiatry.

